# Conditions for success in introducing telemedicine in diabetes foot care: a qualitative inquiry

**DOI:** 10.1186/s12912-017-0201-y

**Published:** 2017-01-13

**Authors:** Beate-Christin Hope Kolltveit, Eva Gjengedal, Marit Graue, Marjolein M. Iversen, Sally Thorne, Marit Kirkevold

**Affiliations:** 1 Faculty of Health and Social Science, Centre for Evidence-Based Practice, Bergen University College, Bergen, Norway; 2 Department of Global Public Health and Primary Care, University of Bergen, Bergen, Norway; 3 Faculty of Health and Social Care, Molde University College, Molde, Norway; 4 Department of Medicine, Section of Endocrinology, Stavanger University Hospital, Stavanger, Norway; 5 School of Nursing, University of British Columbia, Vancouver, Canada; 6 Department of Nursing Science, Institute of Health and Society, University of Oslo, Oslo, Norway

**Keywords:** Telehealth, Telemedicine, Health care professionals, Diabetic foot ulcer, Focus groups interviews, Interpretive description

## Abstract

**Background:**

The uptake of various telehealth technologies to deliver health care services at a distance is expanding; however more knowledge is needed to help understand vital components for success in using telehealth in different work settings. This study was part of a larger trial designed to investigate the effect of an interactive telemedicine platform. The platform consisted of a web based ulcer record linked to a mobile phone to provide care for people with diabetic foot ulcers in outpatient clinics in specialist hospital care in collaboration with primary health care. The aim of this qualitative study was to identify perceptions of health care professionals in different working settings with respect to facilitators to engagement and participation in the application of telemedicine.

**Methods:**

Ten focus groups were conducted with health care professionals and leaders in Western Norway between January 2014 and June 2015 using Interpretive Description, an applied qualitative research strategy.

**Results:**

Four key conditions for success in using telemedicine as a new technology in diabetes foot care were identified: technology and training that were user-friendly; having a telemedicine champion in the work setting; the support of committed and responsible leaders; and effective communication channels at the organizational level.

**Conclusions:**

Successful larger scale implementation of telemedicine must involve consideration of complex contextual and organizational factors associated with different work settings. This form of new care technology in diabetes foot care often involves health care professionals working across different settings with different management systems and organizational cultures. Therefore, attention to the distinct needs of each staff group seems an essential condition for effective implementation.

## Background

People with diabetic foot ulcers need frequent consultations with health care professionals in specialized hospital care. Telehealth technology, often labeled as telemedicine [1], can be helpful in meeting these follow up care needs [2]. Telemedicine supports distant interaction between health care professionals in primary and specialized care, thereby reducing the need for patients to visit the hospital [3], and the uptake of this kind of technology to deliver health care services at a distance is expanding [4]. Nevertheless, adoption of telemedicine in clinical practice is notoriously slow [5, 6], in part because of its complexity when introduced into a heavily burdened clinical practice work situation. Furthermore, problems have been identified in scaling up smaller telemedicine initiatives into wider health care service system redesign [7]. Developments in the technological field have grown more rapidly than has our knowledge about how its organizational and contextual aspects influence how we deliver health care [8]. Evidence to support understanding and addressing these factors is essential to ensuring that telehealth care services are both effective and appropriate for patients [9]. In this evolving telemedicine technology context, a systematic review by Brewster and colleagues in 2014 pointed out that the acceptance of new technologies by health care professionals, as well as their participation and engagement in introduction and implementation, is a vital component of their ultimate effectiveness [10]. However, as Mair and colleagues observed in a another systematic review in 2012, less priority has been directed to date on understanding barriers and facilitators to successful application in relation to involvement and engagement of the health care professionals [11]. Although there have been significant numbers of studies addressing the technology itself and its feasibility, few have focused on understanding the health care professional engagement aspect in either specialist health care or primary health care settings [12]. Interventions that are tailored to increase conditions for success when applying telemedicine across settings might therefore help improve this modality of professional practice [13].

This qualitative study was conducted in alignment with a large cluster randomized controlled trial which was designed to investigate clinical outcomes associated with telemedicine follow-up care for people with diabetic foot ulcers in outpatient clinics in specialist hospital care in collaboration with primary health care (home- based care services) [14]. Qualitative investigations aligned with randomized controlled trials can assist in providing in-depth insights into the processes at play and that may explain the observable effects (or lack thereof) in complex interventions [15, 16], including the implications of the intervention for the actual health care setting and the manner in which new technologies change care delivery patterns [17].

The national health care service system in Norway is financed by taxation, under the principle that all legal residents have equal access regardless of socioeconomic status and area of residence. However, national evidence-based practice guidelines do not specify whether patients with foot ulcers should be enrolled in orthopaedics, dermatology or endocrine outpatient follow-up clinics, or followed by primary health care. Thus, in the Norwegian context, usual follow-up procedures for diabetic foot ulcer can differ depending upon the arrangement of specialty outpatient care options. In most instances, patients have been followed-up by specialized hospital care (outpatient clinics) supported by primary health care (home-based care services) with limited communication between those forms of health care. The aim of this qualitative study was to identify what health care professionals in distinct staff groups perceived as essential conditions for effective implementation of telemedicine as a new health care technology in diabetes foot care.

## The intervention

The general field of telehealth includes a wide range of technology applications, often including computers and mobile phones [4]. The intervention in the current study consisted of an interactive telemedicine platform comprised of a web based ulcer record combined with wound assessment images and text sent via mobile phone or computer from primary health care providers to specialist health care providers (ClinicalTrials.gov: NCT01710774). Images were taken using the camera function of a standard mobile smartphone dedicated to this purpose, and there were no differences in equipment used across sites. The health care professionals in specialist health care contacted the nurse in primary health care in case of uncertainty or if text or images were not received on a weekly basis [14].

For patients included in the intervention group, this constituted a change in the treatment routines for their diabetic foot ulcer, reducing reliance on outpatient consultations in specialist hospital care and replacing them with local follow up by registered primary care nurses who were supervised by health care professionals (mainly but not exclusively nurses) in specialist health care. Primary care nurses used the technology and performed wound care mostly in patients` homes, but for some patients this care took place in a nurse-led primary care clinic or at a general practitioner’s office. The key ingredient in the intervention was the close integration between health care levels with the use of telemedicine equipment to transfer images of the foot ulcer from home care nurses to specialist health care professionals in hospital outpatient specialty services. Nurses performed wound care in accordance with the specialist’s assessment and were required to initiate contact with the clinic should the ulcers exacerbate regardless of prior agreements. In addition to the primary care follow up, patients in the intervention group were seen approximately every sixth week in the outpatient specialty clinic. Those not randomized to the intervention group were followed up in a standard manner in the specialist hospital care outpatient clinics with consultations, usually every second week. When a patient participated in the intervention group, the nurse in charge in the community (home based care service), a nurse at the nurse-led primary care clinic or a nurse at the general practitioners office was contacted by the specialist hospital care outpatient clinics [14].

Health care professionals involved in the intervention received basic training in the use of telemedicine. The training consisted of a review of written information about the study and study procedures together with a hands-on training session that focused on use of the web-based ulcer record as well as the mobile phone. Beyond this basic training, a more advanced level of training with respect to the management of the intervention processes was provided to a small group of health professionals in specialist health care involved in clinical leadership positions who were intended to function as resource persons for the remainder of the participants. The training sessions also presented an opportunity for the visiting health care professionals and hospital staff to meet and get to know each other prior to cooperating on the project. Health care professionals from primary health care were also offered the opportunity to observe consultation as conducted in the hospital outpatient clinic. As the intervention continued, nurses in the hospital outpatient clinic were communicating with the home care nurses at least once a week, and could also communicate with them on an ongoing basis [14].

## Methods

An applied qualitative research strategy known as Interpretive Description was used in this study. Interpretive description is an inductive approach inspired by grounded theory, naturalistic inquiry, ethnography and phenomenology [18]. The hallmark of Interpretive Description is the integration of knowledge development in relation to clinical phenomena, such that health care professionals can obtain new insights informing the clinical field [19].

### Participants

The study sample for this project was comprised of two groups: 1) point-of-care professionals (primarily nurses, but also including other providers) working in either primary/home based care or in specialist hospital outpatient settings, and 2) registered nurses in various clinical leadership roles in primary/home based care or hospital outpatient settings. The point-of-care health care professionals worked either in specialty outpatient clinics located in two Western Norway hospitals or in a primary health care context in the municipalities (municipality districts or clusters) associated with that hospital’s health authority region. Sampling was purposive, including seeking variation in health care occupation, and ensuring inclusion of health care professionals with some experience in how to use telemedicine. Invitations to participate in focus groups were sent by e-mail to all of the health care professionals who had experienced some level of telemedicine within the clusters in the intervention group (*n* = <40). As a result, a total of 34 health care professionals from seven home based care services, one nurse-led primary care clinic, one general practitioner’s office and two specialty outpatient hospital clinics responded and participated. Most of the health care professionals were registered nurses (*n* = 24) or clinical leaders (*n* = 5); the sample of direct care providers also included one nurse assistant, two podiatrists and two physicians (Table 1). The majority of the study participants worked in primary health care (*n* = 21). The original plan was to interview all participants twice with a 6-month interval to find out more about the process they undertook when using telemedicine. However, it turned out that the participants had less experience than assumed after 6 months because fewer patients were recruited in the cluster randomized controlled trial than expected. Therefore, additional participants who had experience with the ongoing intervention into the second round of focus groups were invited to participate.

**Table 1:** Staff participants working site

Working site	Number of participants
Nurses in home based care services	18
Nurse-led primary care clinic	1
Nurse assistant in home based care services	1
Nurse in a general practitioners office	1
Nurses in outpatient clinics	4
Nurses in various clinical leadership roles	5
Physicians in outpatient clinic	2
Podiatrists in outpatient clinic	2

The sample reflected some diversity in education and experience. Eighteen of the nurses had completed advanced nurse education such as wound care, diabetes specialist nurse, intensive care nursing education, and the physicians were specialists in endocrinology. All but one were female, and the participants’ mean age was 47 years (range 24–64). They ranged in wound care experience from 1 to 30 years. Those working in home-based care service were in rural districts. Some of the participants were in the initial stages of introducing telemedicine in their work because of their participation in the ongoing cluster RCT. There was also a range of work experiences related to telemedicine; those working in specialist health care were more experienced in using telemedicine than those working in primary health care due to their more frequent use of the web based ulcer record system.

### Ethical considerations

The Regional Committee for Medical Research Ethics (2011/1609/REK vest) approved the study protocol. In keeping with local conditions for ethical approval in studies involving health professionals, confirmation that they had received written information about the study was obtained in lieu of written informed consent. The participants were assured that their participation was voluntary, and that it was fully possible to withdraw from the study at any time without consequence. Their anonymity was preserved by using numeric signifiers instead of names in all transcripts and notes.

### Data collection

Data was collected from ten focus group interviews during 2014 and 2015. Focus groups were found to be a relevant method for exploring the participants experience in a collaborative environment (Krueger & Casey, 2009). The first author served as moderator with the second author as co-moderator in these interviews, and neither was associated with the telemedicine intervention. The size of the focus groups varied between three and seven participants.

The focus groups were generally held in the professionals` work sites, but for the convenience of the nurses, some focus groups from primary health care were held in a meeting room in the hospital. We chose to hold separate groups for participants from primary health care and specialist health care. There was also one focus group with only clinical leaders. This was done to avoid a possible problem due to the difference in experience in using telemedicine, assuming that this could cause some of the participants to hold back on their thoughts in a group discussion format. It was also assumed that mixing the health care professions within their own working site could facilitate an exploration and discussion on different perspectives within their work context.

The length of the focus group interviews ranged from seventy to ninety minutes. A digital voice recorder was used to audiotape the interviews. A thematic interview guide was developed, including open ended questions designed to ensure as full as possible coverage of the topics related to our study aim. The scope of these questions was informed by previous research on telemedicine as well as important factors known to influence quality improvement (structural aspects, care processes and outcomes of the new way of working). The set of guiding questions aimed at capturing the participants` experience of the planning process, applying telemedicine in their care and the changes they perceived in relation to being introduced to telemedicine (Table 2) [14]. During the focus groups interviews, health care professionals were also encouraged to raise other issues that they considered important in relation to conditions for success in enacting telemedicine in diabetes foot care.

**Table 2:** Guiding questions for focus groups

Guiding questions for focus groups	
• Participants` experiences of using telemedicine, and how it was organized where they work • Participants` experiences in using telemedicine as a new tool in documentation and communication • Participants` experiences in communication and collaboration between outpatient clinic (physicians, nurses and foot therapists) and nurses in home care through tele-communication, and among professions • Changes in their competence in caring for people with diabetes foot ulcers during the intervention	

### Analysis

Interpretive Description methodology served as a guide for the process of analyzing the data [18]. Gathering and analyzing the data occurred concurrently. Early data analysis was conducted immediately following each focus group interview in order to inform and guide the next one. This process helped to compare elements in the data throughout the data collection and analysis process, using a constant comparative approach. Notes were taken during all focus group interviews by the co-moderator, and after each interview the moderator and co-moderator talked through their impressions with respect to what had taken place. All interviews were transcribed verbatim. Immersion in the analysis was achieved by reading and rereading the transcripts to become familiar with all features of the data. Key verbatim segments of the transcripts were extracted and collated for ongoing interpretation. Questions repeatedly asked during this process included: What is seen here in this data? What is not seen? What is going on here? What does it mean? Through this ongoing iterative process, a sense of the whole was developed, from which the body of transcribed material was more formally coded and organized.

The coding process started inductively, using open-ended codes. In accordance with the Interpretive Description approach, data segments were grouped in a manner that was as broad-based as possible to avoid premature closure of our interpretations. The material was independently coded, followed by a discussion around the codes to achieve consensus about the patterns in the material at each stage of the analysis. Through this coding process, groupings emerged that helped organize the data. As the team viewed the collected data and the analysis of it in process, the tentative initial groupings enabled a consideration of the various patterns and variety within those patterns across all the interviews. As possible relationships between these patterns became more apparent, the analytic process was concluded by conceptualizing findings in a manner that optimally illuminated the facilitators to engagement and participation in the application of diabetes foot ulcer telemedicine from the perspective of these health care professionals.

## Results

Throughout the accounts of various facilitators regarding the use of telemedicine in the care of patients with diabetic foot ulcers, it was observed that health care professionals were highly enthusiastic about the idea itself. Nevertheless, they encountered challenges in the process of the telemedicine intervention, at least in the initial phase that was followed for this study. Exploring these challenges in-depth, it was possible to identify four key conditions perceived by the health care professionals involved to be crucial to a successful telemedicine care delivery experience; user-friendly technology and training, a telemedicine champion, committed and responsible leaders and effective communication channels at the organizational level.

### User-friendly technology and training

The health care professionals in this study emphasized the importance of having a user-friendly technology upon which to rely when using telemedicine. After the initial phase, in which they noted some minor difficulties with the mobile phone, passwords and the web ulcer record, the technology seemed to function very well. Many of the health care professionals experienced the technology as easy to learn and use, and this facilitated the intervention for that group. As one explained it, “The technology must be easy to use, and it is. If it takes a lot of time and attention it would make us more reluctant to use it.” Opinions similar to this were common across the focus groups with both primary health care and specialist health care professionals. They frequently emphasized the need for technology that actually helped them in their work without being too time consuming: “One must remember that using this technology is one of many tasks during our day.” There was also agreement on recognition that the telemedicine technology was improving all the time. Participants across all of the focus groups highlighted the importance of being asked and listened to when adjustments were made with the technology.

To learn to use the technology, the health care professionals in the intervention arm in primary health care were invited to participate in an early meeting with the patient when wound care was being performed in the hospital outpatient clinics; however, in some cases, this aspect of the intended plan seemed to slip away as time went by. This led to some differences in how they experienced the training in using the technology and what that training consisted of. In contrast to the health care professionals in specialist health care, those working in home based care services seemed to have more varied experiences in the training for using the technology. Over time, some experienced having to manage the training of their new colleagues in using the technology, and help them perform the wound care expected as part of the intervention. The following example illustrates the frustration they experienced when this occurred:

One of my colleagues received training in using the technology from me. She chose not to use the computer when reporting to the outpatient clinic. She used the smart phone because she had problems with the password on the computer I think. She had many problems in the beginning,…She told me later that she was frustrated and wanted to quit.

Some of the health care professionals in the home based services also experienced the training to be unsystematic. They found this challenging, because they felt they were given more responsibility than expected. In such situations, they expressed the desire for access to more training as needed over time because, when they were not using telemedicine, they forgot how to use it. However, even with gaps in systematic training, the health care professionals generally remained enthusiastic, because they clearly saw the potential benefit for the patients.

### A telemedicine champion

Although the health care professionals generally described the use of the new technology in a positive manner, they also emphasized the importance of having someone close to them who could facilitate the telemedicine intervention. The health care professionals in the outpatient clinics felt that some colleagues in the intervention were their “leading lights or champions” when using this new technology. They referred to these facilitators as essential to this intervention, and crucial for the telemedicine intervention’s success. Such individuals were described as facilitating the intervention by being professionally updated, engaged in the intervention and performing the practical tasks like maintenance and adjustments in the technology; they seemed highly committed to and enthusiastic about using telemedicine.

The importance of having a colleague who could champion this intervention was described as a prominent success condition among those who experienced it as well as those who did not. The following example illustrates the role played by such a facilitator in their midst:

Well, we have some among us who facilitate it all. They have encouraged us, and when some of us think this is too much work, they have been there with their enthusiasm. This enthusiasm has been valuable to us.

In contrast, it was found that the health care professionals in primary health care in particular were unlikely to have a champion at hand to serve in this facilitator role. Instead, they experienced having to be their own champion in motivating and performing the intervention. This made them more vulnerable to exhaustion in relation to the telemedicine intervention, especially when they were the only one over time or during specific periods in their particular district to be using it.

### Committed and responsible leaders

From the perspective of the health care professionals, it was apparent that a related condition of success beyond colleague facilitators was having organizational leadership within their care systems that was aware of and supportive of the telemedicine intervention. Focus group participants highlighted the need for a committed and a responsible organizational leader to support the conditions under which success was made possible. Their accounts made it apparent that the locations and contexts of their work – such as whether they worked in specialist health care or in primary health care – influenced the likelihood of having a leader appropriately positioned in a role with the ability to support the use of telemedicine in their daily care.

The specialist health care work setting made it possible for the leaders to play a more active role when applying the telemedicine intervention in diabetes foot care. Because they were visible in the outpatient clinics, the leaders actively contributed in different ways. They were conscious of being available when needed for the health care professionals taking part in the intervention. The health care professionals experienced their leaders as supportive and helpful in organizing their everyday tasks in the outpatient clinic so they could perform the intervention as planned. One nurse leader in an outpatient clinic described her experience in this manner: “I help in organizing on a daily basis so everything works out fine. I try to be there if they need me. They are skillful, and it is nice to be a part of this.”

In contrast to the leaders in specialist health care, the leaders in primary health care were unable to provide the same level of direct support, mainly because they did not work alongside the nurses in home based care in the same manner. As home based care nurses work in the homes of their patients across a wide geographical area, they have to work independently and make many decisions on the spot without direct access and support from their leader. Therefore, the leaders in home based care services operated from the sidelines rather than in the more visible role of leaders in the outpatient clinics. The differing organizational structures between these two kinds of settings shaped the distinct roles of leaders and thus their approach towards supporting the intervention.

Despite this difference, many health care professionals in home based care services participating in the telemedicine intervention experienced their leaders to be a positive factor in the use of telemedicine, even though they did not engage in it directly. Some of them characterized them as “leaders on the sideline.” As one explained: “In my municipality it is not organized at all. There is no guarantee that I will be sent to a patient involved in the intervention. I have to tell my leader to send me there.” Another member of that focus group quickly responded: “It is the same where I work, I have to remind my leader to send me to the right patient.” Clearly this was not the case in every municipality, as some experienced their leaders as mindful of which nurse to send when patients were involved in the intervention: “In our place the leader makes sure that the health care professionals involved in the intervention visit patients involved in the intervention.”

Even where health care professionals had to organize and manage the telemedicine use on their own, they could still feel some support from leaders when they experienced their attitudes toward telemedicine to be positive. However, when unexpected events occurred, with either equipment or patients in the intervention, their experience was that they had to manage it by themselves. Therefore, taking ownership of the telemedicine intervention seemed to be of utmost importance for the health care professionals in the primary health care context. Overall, we also found that the home based care nurses needed to be more highly qualified and autonomous than those in specialist services. In some focus groups in the primary care context, we encountered reports such as this: “I do not think they (the leaders) know what we are doing when we are using telemedicine.” Another study participant explained: “There is an interest for this intervention by the leaders, but they are not so interested in knowing more about the intervention. My leader is pleased with the fact that I am handling it all.”

Among the health care professionals in home based care services, some concerns were expressed about the vulnerability of the intervention with respect to overall leadership. In particular, where there were limited numbers of health care professionals involved in the intervention, it could seem that no one was assuming full responsibility. Some of these health care professionals expressed the view that their leaders did not know what they were dealing with on a daily basis when using telemedicine, and they had to organize their working day as best as they could. When there were no leaders fully engaged in the intervention, it seemed most vulnerable. This was especially the case when those involved were unable to participate in the intervention, such as when care staff were sick, on maternity leave or holiday, or there was a change in the job situation. As one explained: “It has been a challenge when I have been on a holiday. No one is responsible then. No one follows up these patients in particular.” Another similarly commented: “I wish we were two health care professionals involved in the intervention because my experience is that when I am away no one follows up messages from the outpatient clinic.” The effect of such situations was that the intervention was not carried out as intended, and pictures were not taken and reported as planned. Because performing wound care and documentation within the intervention was more time consuming than usual care, some of the health care professionals had to argue for spending more time with patients. They interpreted this not as a lack of enthusiasm from their leaders, but rather as a lack of involvement in the intervention because they trusted the health care professionals to perform the intervention as planned.

It was noted that some participants in a focus group interview with the leaders in home based care services described a similar limited degree of involvement in the intervention to that which the health care professionals in that setting had experienced. For example, some were very uncertain about what the elements the intervention actually consisted of, and did not know what kind of training the health care professionals had in relation to the intervention. They strongly felt that they had delegated responsibility for performing the intervention to the selected health care professionals in the municipality, and seemed satisfied with the situation. A comment by one leader illustrates: “Some nurses were given this responsibility. They have taken this responsibility, and have control. I am here on the sideline, but I find it exciting.” Another agreed: “It is the same in our municipality. Those nurses involved in the intervention have total control, and they inform me when needed. I just follow them from the sideline.” In this manner, the leaders relied on the competence of the health care professionals and made them responsible for carrying out the intervention. While they did not feel that they had facilitated anything for the health care professionals, they stated that they were very positive with respect to the intervention. For these leaders, the intervention was not perceived as time-consuming at all. They reflected primarily on the positive aspects of care that this intervention contributed to, such as more competence in wound care within all health care professionals in primary health care.

### Effective communication channels at the organizational level

The final condition for success observed in this study had to do with effective lines of communication within health care organizations. In primary health care there are leaders at several levels. The leaders in primary health care felt that they missed information about the intervention, and had some concerns that information from “higher up” in the system did not reach them at all. Furthermore, they did not know who to talk to in order to get such information. A typical remark in the focus group by the leaders was: “I do not receive much information. I do not know if there is any key person to ask either. If there are any meetings for some key persons, they do not pass on information to us.” In addition, opinions like this were expressed: “If leaders higher up in our system know something they should inform us. We do not know if this project is still running.” Several felt that this intervention should have been more strategically driven by leaders above them who were prepared to keep them better informed. Lack of information impeded the ability of the leaders to follow up on the implementation of the telemedicine intervention and fully support their staff in the implementation process.

Effective communication was also influenced by the fact that, in each municipality, there were only one or two selected health care professionals in home based care services involved in the intervention. From the perspective of the specialist health care professionals involved in the intervention, this facilitated communication in that they found it easier to have to cooperate with only a few individuals in primary health care. As one explained: “We do not want more people to be involved. It is much easier to communicate with a few.” In contrast, those involved in the intervention in the primary health care experienced this to be a problem because of the vulnerability in there being too few involved when some of them could not participate for various reasons. Then communication sometimes broke down or was delayed, which might threaten the continuity of the foot ulcer follow up care.

To summarize, identifying these four distinct but intersecting key conditions for success provides additional layers of knowledge about the complex processes involved in introducing telemedicine in diabetes foot care.

## Discussion

The findings of this study reflect conditions for success from the health care professionals’ perspective in the initial phase of introducing telemedicine in diabetes foot care. A set of distinctive but interrelated conditions have been identified that seem central to a positive experience for the health care professionals involved and therefore for the success of the intervention. These conditions seem linked to one another, reflecting variations in the contexts in which the health care professionals work as well as structural and organizational conditions of the telemedicine intervention. The importance of having technology that is easy to learn and use, which was identified as one of the main conditions in this study, has also been described in other studies [7, 10, 11, 20]. The need for a user-friendly technology, such as was highlighted both in specialty outpatient clinics and in primary health care, was crucial on the basis that early negative experiences could make them reluctant to use the technology. Continual improvements in the technology, such as 3D imaging systems to image and measure the wounds, might well facilitate the use of telemedicine when the new technology is ready and available [21], as well as support in using the technology.

Training in using the technology in the initial phase, a factor that was crucial for the health care professionals in this study, has also been reported as a success factor in other studies [7, 10, 11, 20, 22]. However, the health care professionals in the current study found that they required training not only in the initial phase, but also later on in the process. The absence of this ongoing training contributed to a sense of insecurity about using the technology. In some cases, especially in the primary health care context, this insecurity seemed to reduce their enthusiasm for telemedicine and therefore hindered its use. Similarly, in a study from United Kingdom, it was found that ongoing training served as a key facilitator in this process [20].

The importance of new technology is not always visible for the health care professionals, and their enthusiasm might therefore be correspondingly low. In this study the health care professionals’ enthusiasm in using telemedicine from the beginning seemed especially important when they encountered equipment limitations. The primary source of that enthusiasm was information they had received about the potential added value telemedicine could contribute to care for their diabetes foot ulcer patients. They experienced that it provided a better health care service to their patients and higher quality in the care.

It has been recognized in the literature that reservations by health care professionals about using this new technology may hinder the uptake of it [20]. Based on the current findings as well as confirmatory evidence in the literature, it seems obvious that having a robust system involving user-friendly technology and a good support and training system available at all times would be the ideal in implementing telemedicine [10, 20]. It might be an option in the future to use electronic devices, such as web-based courses, to facilitate ongoing training and support for the health care professionals during the implementation process, including in the context of long-term use of the technology. Thus, early consideration of these kinds of contextual and organizational factors is needed for a successful adoption of the technology.

In the current study, the lack of robustness of the intervention plan was found to be more conspicuous in the primary health care context than in the specialist settings. In that context, the health care professionals were more likely to work alone with the new technology and were therefore more vulnerable to unpredictable situations. The infrequency of their use of the technology because they encounter fewer patients in the intervention group made confidence in using telemedicine more difficult to achieve. The current findings also stress the importance of having nurses in primary health care who are highly qualified, autonomous and self-reliant due to their independent role in that setting.

Having a competent professional colleague was an especially important condition for success according to the participants in this study. Professionals in both the specialist health care and the primary health care contexts emphasized the impact of the facilitating role that some of their colleagues had assumed. Such individuals motivated ongoing enthusiasm and willingness to use the technology among their colleagues even when problems occurred. This suggests that it would be important to take into account the presence or absence of a potential champion when planning an intervention such as this. Although the potential value of champions has been noted in earlier studies [7, 20, 22, 23], this condition for success has not featured strongly in prior accounts. Given the diversity within our sample with respect to this condition, it would seem that this aspect ought to be emphasized in future initiatives so the health care professionals in all settings can benefit from the champion aspect. Thus, one question arising from these results is whether it will be possible to identify individuals with the capacity to serve a champion role and guide them into that role at an early stage. Understanding what motivates champions within such interventions and how best to facilitate their involvement through the entire process seems essential for future implementation projects.

An observation arising from other studies is that using telemedicine necessitates a change in organizational thinking around collaboration and health care professional roles within a given work setting [20, 22, 24]. In the current study, having organizational leadership within the care system that was supportive and aware of the telemedicine intervention was an important condition for success. These findings suggest that implementation plans for such projects should not neglect the importance of having deep knowledge around the kind of work context or culture into which the new technology is being introduced. It may be that different strategies will be required for the introduction of telemedicine approaches to diabetes foot care within different contexts. Technical devices might be received differently in different work settings [17]. The current findings clearly demonstrated different issues arising in the work setting of the primary health care context relative to that of specialist health care. Specifically, it was noted that there was an increased requirement for attention to leadership and effective communication channels within the organizational level in the primary health care context. This aspect would likely be of particular importance to any implementation of telemedicine on a larger scale. Beyond this essential collaboration and communication, however, these findings also suggest that having health care professionals who are capable of working independently and championing their own work may be a necessity for the successful introduction of these new technological innovations.

### Methodological considerations

One limitation to this study might be the difficulty in gathering the participants for a second focus group interview. Despite attempts to reschedule to accommodate all potential participants, some of the health care professionals were unable to join a focus group for various reasons. There is a possibility that some of those who were missed would be more experienced, and could have provided more variation in degrees of experience. On the other hand it is more plausible that those who are more reluctant did not participate in the focus groups. They could have provided more knowledge related to negative experiences on the intervention. A further limitation to this study might be the use of only focus group to gather data. Given the opportunity to use observational methods, it might have been possible to benefit from richer data with respect to what was going on in the actual care context into which the telemedicine intervention was being introduced.

More research will be needed in the future to explore this implementation processes in detail. Both observational studies with health care professionals and individual interviews with patients would expand our knowledge base. There will also be a need for future research into the implications for success when interventions are more tailored to each working context. Nonetheless, on the basis of this analysis from the perspective of a diverse group of health professionals engaging in this innovation in their care of patients, it has been possible to provide useful insights for future efforts in technological innovations in the care of patients with diabetic foot ulcers.

## Conclusions

This study draws attention to the value of careful planning to accommodate the needs of health care professionals using new technologies, recognizing that they often work in different settings with different management systems. Although a study using qualitative methods within a specific regional context cannot yield findings that are formally generalizable, what has been learned about these four key conditions for success in using telemedicine can provide those planning such transformations in health care services with an understanding of possible obstacles and facilitators when introducing new technology in a range of health care settings.
